# Supplementary figures and images for: TMT-Opsins differentially modulate medaka brain function in a context-dependent manner
Source: PLoS Biol. 2021 Jan 7;19(1):e3001012. doi: 10.1371/journal.pbio.3001012 (PMC7837489; doi:10.1371/journal.pbio.3001012)

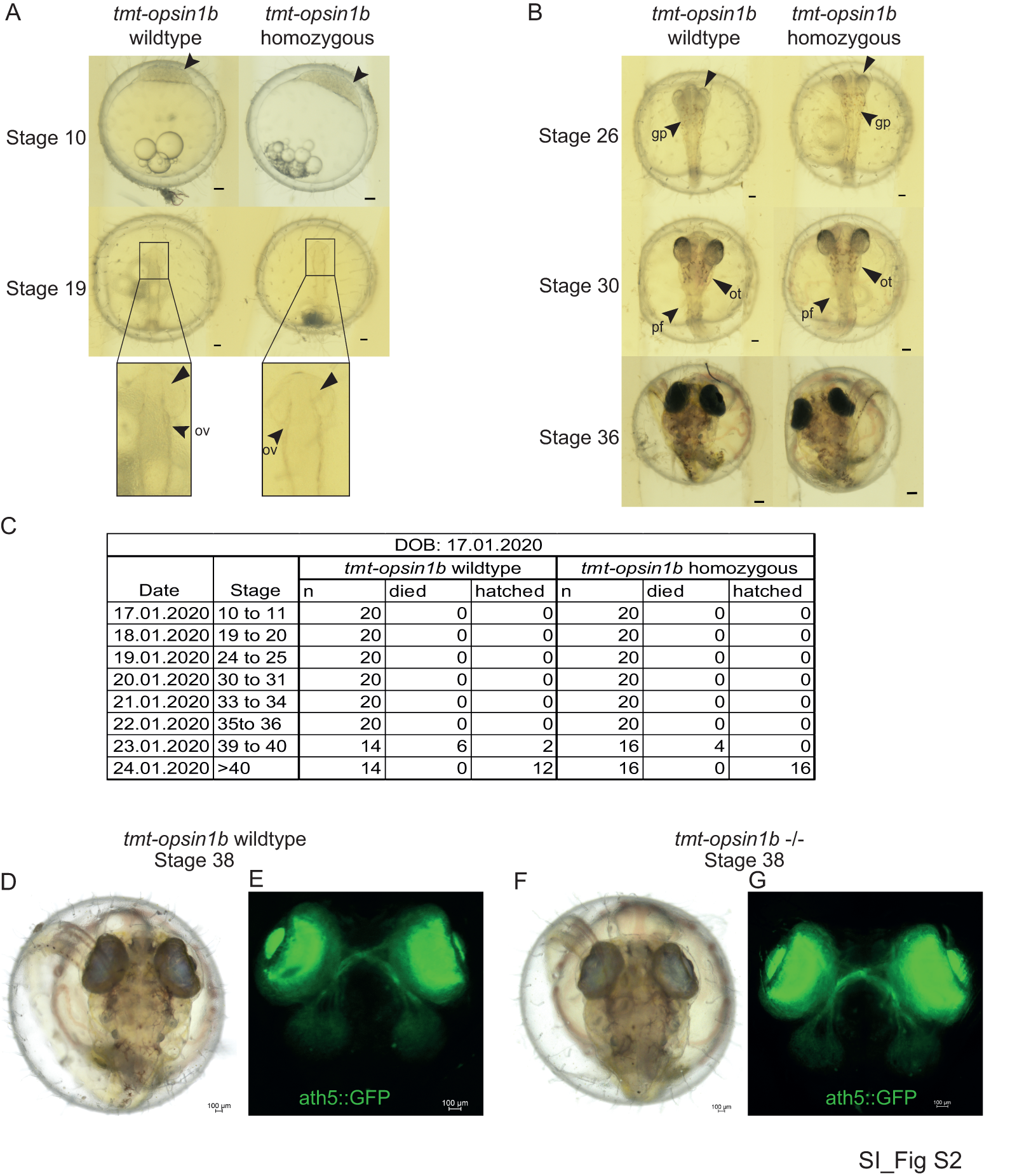

Supplement: S2 Fig — (A, B) Embryonic staging and development using morphological criteria for wild type and tmt-opsin1b−/− mutant according to [33]. Some representative details: Stage 10 shows for both wild type and mutant a thick blastoderm, with smaller inner cells. At Stage 19 both wild type and mutant show the appearance of otic vesicles (ov), as indicated. Furthermore, a groove in the optic lobes can be observed. With Stage 26, both wild type and mutant show that the choriodea start to cluster, as indicated by the arrow, and the guanophores (gp) are clearly visible. At Stage 30, the pectoral fin (pf) is apparent for both, as well as the otholits (ot). For Stage 36, the tip of the tail reaches the otic vesicle and the guanophores are now distributed from head to tail. Scale bar: 500 μm. (C) The table shows the total number of embryos analyzed, with representatives depicted in (A). Both wild type and mutant have an n = 20. (D–G) Representative images of wild-type fish and tmt-opsin1b−/− mutants shortly before hatching; GFP expression in the fish’s retinal ganglion cells shows no noticeable difference in axonal projections. Scale bars: 100 μm. (TIF) [file pbio.3001012.s013.tif]

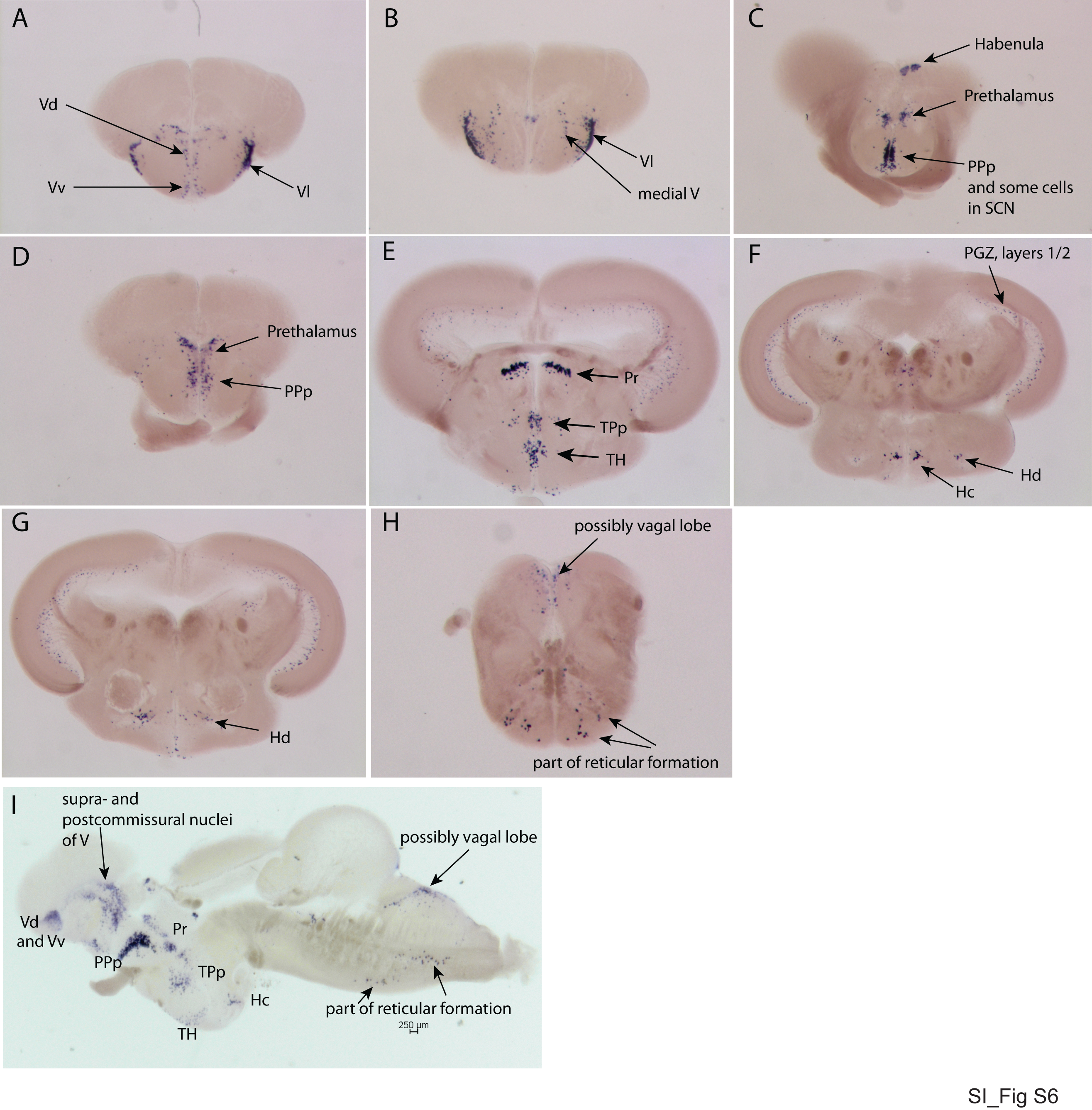

Supplement: S6 Fig — Anatomical annotation of sst1b+ cells as revealed by in situ hybridization in the adult medaka fish brain, coronal/cross (A–H) and sagittal (I) sections. Abbreviations: Hc, caudal hypothalamus; Hd, dorsal hypothalamus; PGZ, periventricular gray zone; Pr, pretectum; PPp, posterior parvocellular preoptic nucleus; SCN, suprachiasmatic nucleus; TH, tuberal hypothalamus; TPp, periventricular posterior tuberculum; Vd, dorsal nucleus of ventral telencephalic area; Vv, ventral nucleus of ventral telencephalic area; Vl, lateral nucleus of ventral telencephalic area; V, ventral telencephalic area. (TIF) [file pbio.3001012.s017.tif]

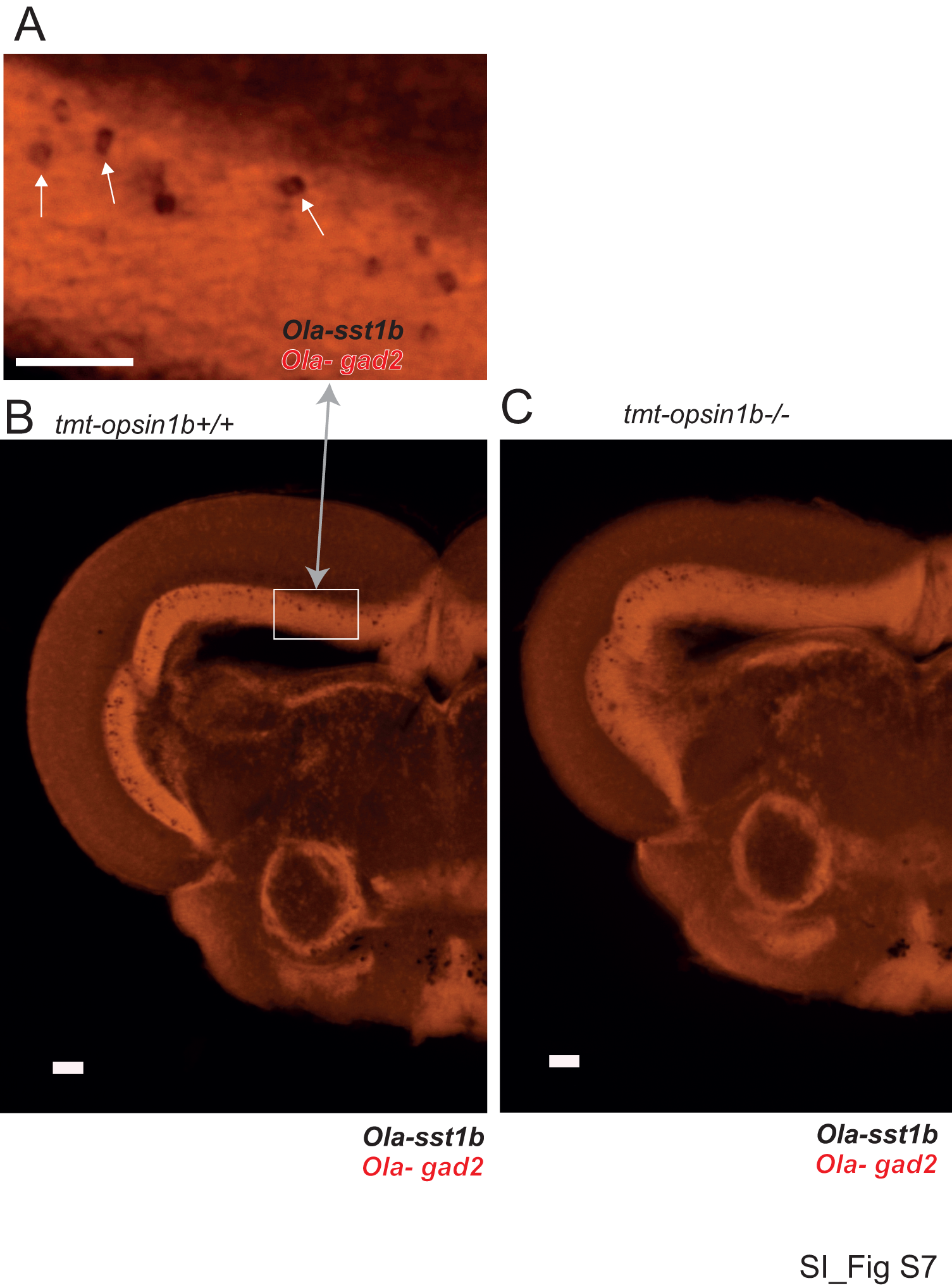

Supplement: S7 Fig — (A) sst1 co-expression with gad2 in tectal neurons (arrows). (B, C) Representative images of in situ hybridizations for sst1b and gad2 on coronal sections of the midbrain from tmt-opsin1b wild-type (B) and tmt-opsin1b homozygous mutant (C) fish document no difference between wild-type and mutant fish. Red channel: gad2. Scale bars: 200 μm. (TIF) [file pbio.3001012.s018.tif]

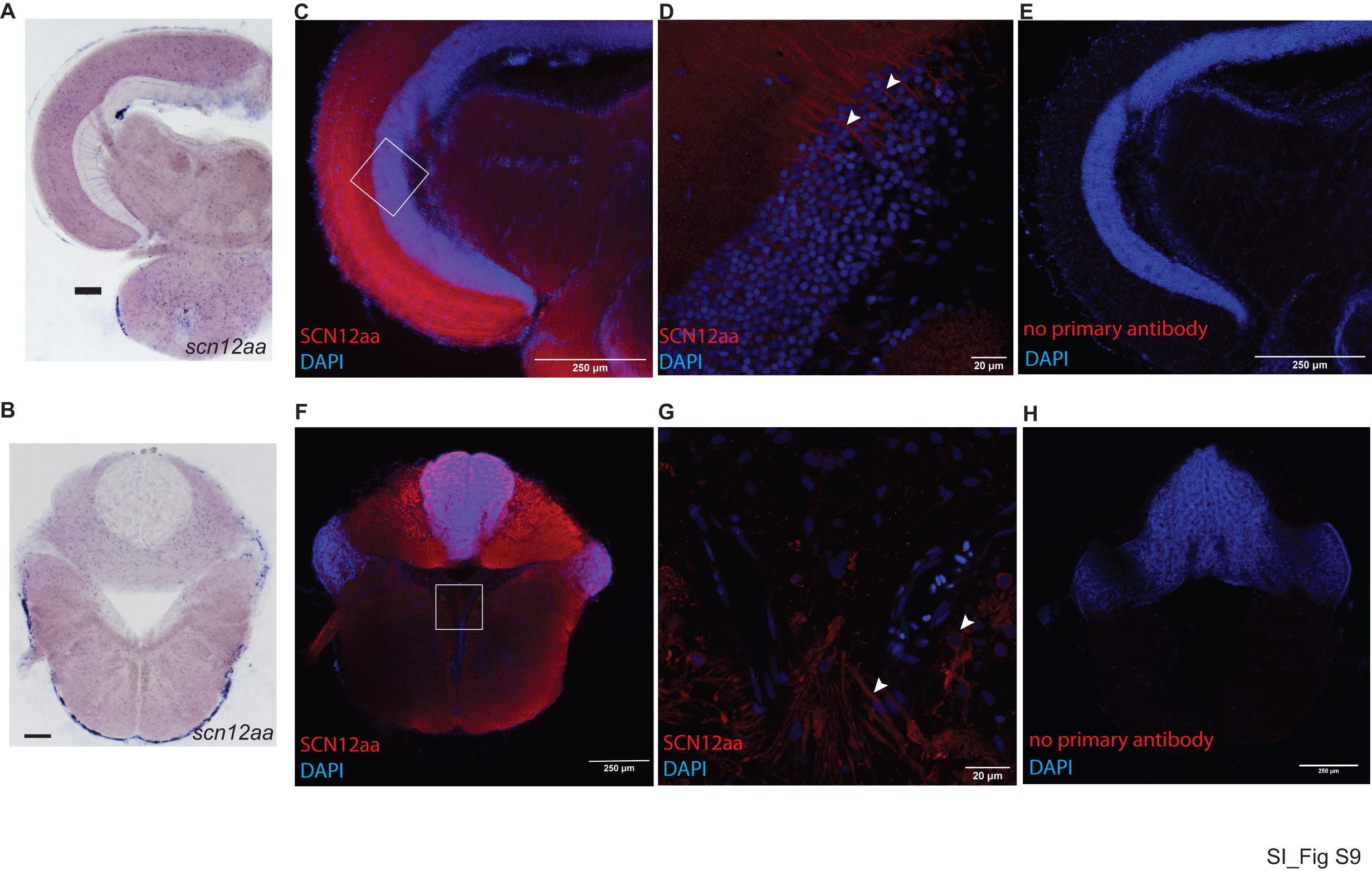

Supplement: S9 Fig — (A, B) In situ hybridization for scn12aa performed on coronal sections of midbrain (A) and hindbrain (B) from Cab wild-type fish. Scale bar: 50 μm. (C–G) Confocal images of brains stained for Scn12aa, using an antibody raised against Hsa-Scn5a (red) and DAPI as nuclear counterstain (blue) on coronal sections of midbrain (C, D) and hindbrain (F, G) from Cab wild-type fish. White box: region corresponding to the 40× magnification. White arrowheads: representative positively stained neuronal projections and cell bodies. (E, H) Control slices with equal treatment as for (C, F), but no primary antibody added. (TIF) [file pbio.3001012.s020.tif]
