# Supplementary material for: TMT-Opsins differentially modulate medaka brain function in a context-dependent manner
Source: PLoS Biol. 2021 Jan 7;19(1):e3001012. doi: 10.1371/journal.pbio.3001012 (PMC7837489; doi:10.1371/journal.pbio.3001012)
Supplement: Attachment — Submitted filename: FontinhaZekoll et al_Response_to_reviewers.pdf [file pbio.3001012.s023.pdf]

We would like to thank the reviewers for the constructive comments. They certainly helped to improve the manuscript, both experimentally, as well as conceptually. We added substantial amounts of additional data and revised the text.
A brief note on the delay in providing a revised version: We initially had a dysfunctional behavioral machine, for which it took the company months to solve the problem. Furthermore, as many labs around the globe, we were hit by a complete shut-down and subsequent slow re-opening, with some operations (like the access to microscopes) still being restricted.
Please find our detailed responses below:

Reviewer #1: This is a very clear and easy to follow paper by Fontinha and colleagues. Since there are ~40 opsins with likely redundant and even, as evident here, opposite roles, the idea to test the effect of individual opsins is quite heroic, and it not surprising that most of the effect are subtle, yet significant. The authors present very interesting results but missing much on providing mechanistic explainations.

We thank the reviewer for the overall positive assessment of our approach.

I have a few comments and question:
There are a number of variables that may affect the results of the avoidance test and should be considered and mentioned:
1. Time for acclimation to the chamber - 5 minutes may be too short. Was this ever tested before?

We understand the reviewers concern, but would like to point out that we used an established avoidance assay for fish and closely followed the published and accepted paradigm as done for zebrafish larvae (see Barker AJ and Baier H, 2015, doi: 10.1016/j.cub.2015.09.055). There is no evidence that zebrafish and medakafish larvae need to be treated differently. While neither previous studies nor we systematically tested different habituation times, we observed that the fish swam normally within 1-2 min and even this shorter habituation time was sufficient to allow for robust, reproducible behaviors across many experiments (even between different labs). This is consistent with a similar visual avoidance paradigm in tadpoles, for which the animals were given only 30s of accommodation time before the stimulus presentation (see methods section in: Dong W et al, 2009, https://doi.org/10.1152/jn.90848.2008).
Thus, we think that by introducing a 5min habituation time- which is longer than in the original and well-established zebrafish larval and xenopus *tadpole assays- makes our analyses at least as robust and standardized as the previously published and accepted experiments. To accommodate for this comment of the reviewer, we now added half a sentence to the methods section, which now reads "The fish were allowed to accommodate to the chamber for 5 minutes before the beginning of the trial, which is longer than in previous similar assays in zebrafish (Barker and Baier, 2015) and Xenopus (Dong et al, 2009), and by this likely ensures a robust standardization across different experiments."*

2. The light intensity in natural environment of the medaka fish. If it is very different from the intensities used in the experiment, the results may be meaningless.

Again, we agree with the reviewer that this is an important comment to consider. For all of our experiments we were very aware to operate within intensity ranges that are likely realistic for the medaka environment and thus had already provided spectral measurements for all experimental light and growth conditions. Medakafish are reported to preferentially inhabit standing and slow-flowing water bodies such as rice paddies, ponds and agricultural channels, furthermore some species are present in brackish water along the shoreline (Hilgers and Schwarzer, 2019, doi: 10.7554/eLife.46994; Larsen GD, 2015, https://doi.org/10.1038/laban.896). While no exact depth ranges, absorbance coefficients nor underwater light measurements at the natural habitat are published or available in the public domain, an approximation of the intensity range across the day is possible.
We now provide a direct comparison between a measurement at a natural water environment (coastal area) across day-night with all different light intensities used in the different experiments (see Supplementary Figure 3). The depth (10m) of this natural measurement is possibly deeper than the natural medaka preference. It could therefore be argued that we underestimate the light intensity. However, this measurement has been performed in clear water, while rice paddies and ponds are often more turbid, which has big impacts on underwater light intensities. For more detail on this e.g. see Kiang, N. Y. et al 2007, doi:10.1089/ast.2006.0105. Furthermore, this measurement was performed in summer, when sunlight is approximately 10x higher than in spring or winter (depending on geographic position). Furthermore, as mentioned in our introduction, light intensities can differ 1000-fold just based on weather conditions (Posch et al. ISBN-13: 978-3527409464). Thus, we believe that for an approximation, using this published underwater light measurement is valid.
From this it is very clear that all of our light intensities are within the natural ranges, thus our results are clearly meaningful for the biology of the fish.

Although not explicitly mentioned by the reviewer we also want to point out that we are of course aware of the spectral differences in natural environments versus our experimental settings. This concerns in particular the combinations of wavelengths that will likely be relevant for fishes, but also other animals. Here, we can only point out that we move along established experimental paradigms, and that basically no indoor illumination (in the past and present) closely matches the natural illumination (see e.g. Dekens M et al, 2017, doi: 10.1371/journal.pone.0172038 for exact example comparisons). However, we believe that our work nevertheless can help to uncover basic principles of (combined) opsin functions in fish. How this will exactly affect the behavior of medaka in the natural light environment remains to be determined (as this is also the case for all studies using in-door artificial illumination for the study of light receptors in organisms). We thus added a final sentence in the discussion to point this out: "… Future work is needed to not only further delineate these interactions, but to also better understand their significant complexity in the context of natural light habitats and adaptations."

3. The light intensity in which the fish were raised prior to the experiments. If intensity changes dramatically it may have an effect.

The light conditions under which the fish were raised were as similar as we could possibly ensure. It is technically impossible to provide fully identical light conditions to different tanks and dishes. We purposely positioned mutant versus sibling wildtype batches and batches from heterozygous crosses in random, alternating position relative to the respective light sources. We now added this point also as a sentence in the materials and methods section: "Mutant versus sibling wildtype batches and batches from heterozygous crosses were position in random, alternating position relative to the respective light sources while grown prior to the assays. Parental fish grew in as similar fish room light conditions as possible."

Also, what do we see here? Is it the effect of the mutation on preception of light intesity? or the effect of the mutation on enxiety/stress?

We thank the reviewer for the interesting thought. In order to discriminate light effects from potentially generally elevated anxiety/stress levels in the mutant fish, we measured total cortisol in the medaka larvae identically raised to those larvae with which the test under different light conditions were performed. Consistent with our hypothesis that light has an impact on the information processing via Tmt-opsin1b*, we did not find any general elevation of cortisol in mutant fish. These new data are now presented in Fig.2P,Q and in the corresponding result sections of the text:*
"Finally, in order to test if the observed elevated locomotion levels are caused by generally altered stress or anxiety levels, we measured their total cortisol levels We observed no difference between mutant and wildtype siblings at neither young larval nor juvenile stages (Fig.2P,Q), while our positive control, larvae exposed to mechanical disturbances (MD), showed a significant increase of cortisol levels (Fig.2P). This strongly suggests that the difference in locomotion apparent between mutants and wildtype is indeed mediated by the acute light changes involving tmt-opsin1b."

Similarly, it is not clear what is the meaning of the change in behavior under blue light and dark and the age-dependent changes. Could the mutation have a developmental effect (see also below)?

Again, we agree with the reviewer that this is a very relevant and mechanistically important question to address. Already prior to the initial submission, we had checked if there were any noticeable developmental differences, which there were none. We now quantified and extended these data by
1.) doing an exact staging of mutant vs. sibling wt clutches based on the occurrence of developmentally timed morphological criteria
2.) We also obtained a line that expresses GFP in the axons of the retinal ganglion cells (ath5::GFP). We crossed this line to tmt-opsin1b-/- mutants and were able to at least raise the resulting F1 heterozygous fish, which we subsequently incrossed. The resulting larvae, which expressed GFP, were analyzed for any morphological differences in the onset of GFP expression (as a proxy for eye differentiation), axon outgrowth and targeting of the axons into the tectum. Afterwards these fish were genotyped to discriminate between homozygous mutants, heterozygous and wildtype larvae. There was no observable difference between the different

genotypes. This strongly suggests that at least eye and tectal development and wiring are developmentally normal in tmt-opsin1b-/- mutants.
The data for 1 and 2 are now presented in Suppl.Fig.2 and also referred to in the text: "We next tested for noticeable developmental alterations by detailed staging of the developing larvae… ."

3.) We furthermore also analyzed possible co-expressing genes in the adult brain. The candidates were selected based on single cell sequencing analyses of larval (23-25dpf) zebrafish brains (Raj, B et al, 2018, doi: 10.1038/nbt.4103.). For further detailed discussion, see our response to the suggestion with the co-expression raised by the reviewer.
We especially focused on gad2. Again there was no visible difference between wt and *tmt-opsin1b-/- adult fish brains. The new data are presented in Supplementary Figure 7.*

All of our analyses therefore suggest that there is no developmental effect, caused by the mutation. However, it is of course impossible to really prove the absence of any developmental effect. Thus, we phrased our conclusions on these analyses deliberately carefully.

And, the subtle differences in the 48 hr experiment, could these reflect of change in chronotype? A change in chronotype could account for the differences in activity in the early morning. The answer to that, fish need to be followed under constant conditions.

Again, this is certainly an interesting and relevant question. We followed the reviewers suggestion and initially attempted to look for potential chronotype differences (i.e. changes in phase under the light-dark cycle and/or period length under constant conditions) using a behavioral locomotor read-out. However, consistent with the results from a previous paper on the circadian rhythm and clock genes in medakafish (Cuesta, IH et al, Chronobiol Int., 2014, Fig.1, doi: 10.3109/07420528.2013.856316), clear behavioral rhythms under constant conditions are only present in fish groups and even then rather variable. They are certainly too variable to conclude on the absence or presence of differences between wildtypes and mutants.
We thus turned towards the molecular oscillations of the core circadian clock genes and selected per1b *and* reverbB (*Cuesta, IH et al, Chronobiol Int., 2014, doi: 10.3109/07420528.2013.856316) as two representative genes, as these might function in slightly different combinations of core circadian oscillators (at least in analogy to the mechanisms in mouse). qPCR on whole larvae matching the age of fish in several behavioral experiments showed that there is no difference in the circadian oscillations of the two core circadian clock components under light/dark and constant darkness conditions, clearly suggesting that the behavioral and molecular differences observed in the mutants are not due to alterations of the circadian core clock, including chronotypical differences. The new data are now represented in Fig.3E,F and the corresponding text: "Light is an important entrainment cue for the circadian oscillator. We thus next analyzed, if the mutation in* tmt-opsin1b *impacts on the phase or period length… ."*.

The paper describes an effect of tmt mutations on sst1b mRNA levels which was discovered by RNA-seq of mutant vs. wildtype brain regions. Information about this data is missing. For example, how many differentially expressed genes were found? What kind of genes? Was sst1b one of the highest changing genes?

Yes, sst1b (meanwhile also called sst3 *or* cort *in ENSEMBL) was originally uncovered by a RNAseq approach of brain parts. As we were mainly interested in changes in the brain, we focused on transcripts changed there and not in the eye. We now include the RNA-seq statistical result sheets* of *tmt-ops1b mutant vs. wildtype brain regions as Supplementary Data (Supp.Data 1,2). According to statistical recommendations, we choose an FDR cut-off at 0.05, resulting in 3 significantly changed transcripts in the forebrain and 22 significantly changed transcripts in mid/hindbrain. (Please note that, as also mentioned in the manuscript, the mid-/hindbrain part also contains a portion of the posterior hypothalamus, as this is located underneath the midbrain.) Of all significantly regulated transcripts, sst1b is most directly connected with neuronal functionality and behavior (at a middle position, based on its FDR score). We also tried to verify the most top-ranked candidate of both brain part sets (trim55). However, this gene showed a very high variability among different verification sets. The main category of the remaining DE transcripts were spliceosomal RNAs, which might be interesting, but to understand their connection to tmt-ops1b and brain functionality is itself a whole project for the future. Last, but not least, it is common for results of screens (in flies, worms and fishes) to analyze specific candidates from such screens and not the whole list, and we think that given the behavioral phenotypes and* sst1b *being a pre-pro-hormone, this choice is well justified.*

The effect on sst1b is further analyzed by ISH on brain sections. This is a very important analysis. Looking at figure 5, it seems to me that there are less sst1b cells. Could this be a developmental effect? Are there other differentially expressed transcripts that are expressed in the same cell types? The answer may be hidden in the RNA-seq data and other possibly available scRNA-seq data. If the mutation has a developmental effect the results can and should be re-interpreted.

As mentioned above, even using an ath5::GFP line we did not observe any developmental phenotype. Also, there is no evidence for a developmental phenotype from the RNAseq data. As suggested by the reviewer, we also looked for possible transcripts also present in sst1b*-expressing cells, to see if those would be present in places where* sst1b*-expression is absent in the mutant. Since there are at present no scRNA-seq data from medakafish brains or larvae available, we looked in the data available from the zebrafish larval brain (Raj, B et al, 2018, doi: 10.1038/nbt.4103.) for cells positive for the* sst1b *zebrafish ortholog. Indeed, such cells existed in the published lists. We identified four genes that are co-expressed with* sst1b *in zebrafish neurons, have 1:1 orthologs in medakafish and (based on existing expression data or type of gene), a reasonable chance to exhibit a relatively specific expression (in principle: the more specific, the better). Unfortunately, of these four genes we were only able to obtain reliable* in situ hybridization *expression on adult brain slices patterns from two: gad2, submitted under GenBank: MT267535 and pcp4l1, submitted under GenBank: MT239386. The transcripts of both genes showed a highly similar pattern and unfortunately not as restricted, as we would have hoped for. In order to not extend the already complex manuscript with too many further details, we just present the* gad2 *result (but could add the* pcp4l1 *if still wished by the reviewer). We confirmed that* gad2 *is indeed co-expressed with* sst1b *(see Supp.Fig.7A), yet only in the tectum. However,*

overall again there is no indication of any developmental (morphological/expression) difference (Supp.Fig.7B,C).

The time of sampling in the photoperiod experiment is not clear. In page 11 it is stated that 'After one week, all fish were sacrificed between ZT0 and ZT3, respectively (blue arrowhead Fig. 6A)' Figure 6A shows that extending the day in long-day photoperiod doesn't change the time of ZT0. Therefore both groups could be sampled at the same time and the same ZT. Why '…ZT0 and ZT3, respectively'?

We thank the reviewer for making us rethink about this important experimental aspect. We had initially followed the sampling timing of Dulcis, D et al (2013) 10.1126/science.1234152, who had also analyzed a somatostatin-type transcript and had kept the time of the day (i.e.hour) fixed. However, with different daylength this of course then results in different ZTs at which the samples are taken. We then checked in other publications, how comparable samples under different photoperiods were obtained. One manuscript technically close to our study is the analysis of the seasonal regulation of a lncRNA in medaka fish (Nakayama T et al. 2019, doi: 10.1038/s41559-019-0866-6.) When comparing samples between long-day and short-day exposed fish, the authors sampled at the middle of the respective light period. The rationale behind this is that if an analyzed transcript or hormone has a circadian profile (which is indeed often the case), it depends on photoperiod, but the profile will still be regular relative to the light-dark periods (e.g. see Fig.6 in Okano et al, 2020, doi: 10.1038/s41598-020-61877-4 for a good fish example or Fig.3 in Sumová A, et al.2003 JBR for rat). We then first analyzed if medaka sst1b *is circadianally regulated and if there is a phase difference between wt and* tmt-opsin1b *mutants, finding that it is circadianally regulated and that there is clearly no phase difference between wt and mutants (new Fig.7C). We then took the brain samples for comparison between photoperiods in the middle of the respective light-phase (as reasoned above).*
This new sampling strategy revealed three results:
1.) It confirmed that sst1b *in eye, midbrain and (tendentially) also hindbrain is down-regulated under short days.*
2.) However, in contrast to our previous interpretation, this down-regulation is not abolished in tmt-opsin1b *mutants.*
3.) To the contrary, it is very obvious from the data, that the mutants downregulate sst1b *even more than wt. This is most obvious in the forebrain, with no change between long- and short day in wt, but a significant change in the mutant. Interestingly, this is in line with the behavioral phenotypes in juvenile fish and the responses of larvae in the avoidance assay, where environmental light changes/ dot approaches also result in stronger responses in the* tmt-opsin1b *mutants compared to wt.*
These new photoperiod data are now represented in Fig. 7 D,E. Experiments were performed such that all graphs are comparable to each other.

Embedding these new data in the overall observations, we thus conclude that now even more consistently the picture emerges that tmt-opsin1b *is normally required to "buffer" physiology and behavior against environmental changes.*

We would have very much liked to repeat these experiments with eyeless fish as well. Unfortunately, for this we had to ask to extend the number of eye removals in our animal license. Although we submitted the request for this to the ministry already at the beginning of the year, we only received the permission on July, 15th, much too late to still perform the experiments.

We now replaced that entire old Fig.6 with the new data (now new Fig.7) and also changed the corresponding text in results and discussion: "We exposed wildtype fish to two different white light regimes … . ."

Regarding the presentation of the photoperiod experiment results. Are the results presented in 6C and 6D comparable? Are they normalized all together? It is important to know what has changed, was it lack of elevation upon placing in long photoperiod?
Again, the same comparison of inoculated fish vs. untreated fish is important and it is not clear if they were all normalized together. I would suggest that all results presented in figures 6C-E are normalized together.

All points addressed in answer above. (Experiments were performed such that all graphs are comparable to each other as requested by the reviewer. This was also already the case in the initial version of the manuscript.)

Discussion focuses of the photoperiod results and neglects much of the behavioral results. There should be much to discuss if the results are important.

We followed the reviewer's suggestion and substantially revised the discussion section, now also covering a possible tectal circuitry involved in controlling this behavior (as also requested by reviewer 3), the age-dependency of the behavioral phenotypes and its meaning for experiments, but also for an ecological context, as well as more the complex interplay of the analyzed Opsins. Due to the new data we also revised our discussion on the role of tmt-opsin1b *in photoperiodic responses.*

Minor comments:
Introduction, 4th paragraph '….particularly to the gonads' I would suggest to change to 'particularly to the reproductive system'

Changed.

Introduction, 5th paragraph, '….and represents ancestral ciliary-type Opsins'. Please explain what are C-opsins. It is a confusing term given that the c-opsins you are investigating here are not expressed in clasical ciliary photoreceptor cells….

In principle we agree with the reviewer that more explanations would be helpful, especially for readers less familiar with Opsin *phylogeny. However, this aspect is not directly connected to the main message of the manuscript and thus risks deviating the readers from the main thread. In*

order to find a balance between the valid point of the reviewer and our concern of making the text too complex, we now shortened "ciliary-type Opsins" to "c-Opsins" and also quote an additional paper on Opsin *phylogeny and evolution (Ramirez et al 2016, doi: 10.1093/gbe/evw248). This paper also discusses the terms "ciliary opsins"/"c-opsins" in the context of phylogenies and any interested reader can then read on.*

Page 6 second line, '….Fig. 1E,G,H), are statistically….' should be changed to '… Fig. 1E,G,H), became statistically….'

Changed.

Pages 8-9, 'while in larval stages adding the tmt-opsin2 mutant ….. in the same experimental test during juvenile stages (Fig. 2G,H and Fig. 3I,J).' I do not see what is described - the slight effects are not much different from larva to juvenile stages. Am I missing something? And, again, the effect on activity may be an effect on chronotype.

We are aware that with all data from wt and single mutants included, these are relatively complex figures. We now improved two aspects. First, we provide a supplementary figure in which we plot the movement for each genotype for the relevant time points individually in order to make the graphs better visible (Supplementary Figure 5). Second, the differences are very clear in the statistical comparisons. We now added a short statement in the text to guide the reader to this and also added arrows to the most relevant graphs.
We ruled out that the behavioral differences for tmt-opsin1b *are due to any changes in circadian phase or period length, which should be the case if there were chronotype differences. Due to experimental time limitations (Corona-restrictions), we could not perform the same set of experiments also for tmt-opsin2 and the double mutants. However, we think that it is unlikely due to chronotype differences, because the timing of the behavioral maxima (and minima) is still the same, what has changed is the amplitude. A different chronotype normally means that the timing of the maxima (and/or minima) of a diel pattern is altered. This does not appear to be the case in tmt-opsin1 and 2 (and double mutants).*

In qPCRs, please indicate the number of samples.

Number of samples (i.e. biological replicates) are indicated directly in the figures.

Reviewer #2: TMT-Opsins differentially modulate medaka brain function in a context-dependent manner

There is considerable interest in the role of the large opsin family in the regulation of cellular and animal physiology. However, part of the problem in this area is the large number of potential

photopigments many of which have yet to be fully characterised. The authors present some experiments using the TALENs approach to attempt to knock down two members of the TMT family, tmt-opsin1b and tmt-opsin2. I was somewhat surprised that the authors fail to introduce the full complexity of the opsin genome in the teleosts. They investigate two of the three TMT families, with no clear rationale for their specific choice. The authors themselves have previously reported that one of the TMTs is co-expressed with at least one other non-visual opsin. This in itself alludes to some of the issues of ascribing a photopigment function to a single opsin. Thus, if you ascribe a photopigment function to a TMT – it could equally have an accessory function for another pigment, like for example VA-opsin? To assign a specific role to a particular pigment requires additional data. for example the spectral and absolute sensitivity of the system, and here this data is lacking.
The authors attempt to ascribe a specific function to two TMT opsins, but fail to address these opsins in sufficient detail to provide a full or definitive answer. There are also issues relating to the methodology and interpretation. In this regard the current data whilst interesting are still rather preliminary. In short they fail to provide a definitive and clear description of the role of TMT in the teleosts that would be suitable for a broader Biology audience. The study raises important issues that need to be addressed in significantly additional experiments.

We are happy to read that the reviewer shares the view of the general interest in a better understanding of the role of opsins in cellular functions and animal physiology. As he/she points out, there is a large number of these opsins, especially in teleosts. Any functional analysis has to start with individual members and it is just functionally impossible and completely unrealistic to ask for FUNCTIONALLY *covering the full complexity of teleost opsins. Also, as we can clearly show in our study, the combination of knock-outs from even just two members can cause rather unexpected effects, on the molecular, as well as behavioral level. Thus, it will be impossible to understand combined knock-outs without studying individual members. Performing an in-depth functional characterization for more than two Opsins and their combination at once is unfeasible. Also, even with "only" two, we already provide significant novel insight into the role of these opsins and their interplay. Therefore, we think that our approach of focusing on two as a starting point is fully justified.*
But following the reviewer's comment, we now added several referrals to the diversity of Opsins in teleost to the text.
As to the specific choices- we chose to first study the combination of two tmt-opsins, because we have previously (Fischer R et al 2013, doi: 10.1371/journal.pbio.1001585.) shown that tmt-opsins in zebrafish and medaka (including medaka tmt-opsin1b and 2*) most strongly respond to blue light (confirmed independently and in more biochemical detail, including absorption spectra, for medaka tmt-opsin1a and 2 by Sakai K et al, 2015, doi: 10.1371/journal.pone.0141238) and that tmt-opsin1b and 2 are also very similarly expressed, likely partly co-expressed or at least in closely neighboring cells. We were thus curious if this apparent similarity in absorption and expression would reflect itself in a simple functional redundancy.*
It is certainly also interesting to study other combinations of opsins, such as a VA-opsin, but there is less biochemical characterization on medaka VA-opsins *then there is on* TMT-opsins. *Thus, also in connection with existing work, we think that our choice is fully justified.*

Concerning the spectral characterization, in addition to our previous work in tissue culture- which already showed that TMT-*opsin1b and 2 alone are fully sufficient to cause light-dependent responses in cells (which in many publications is taken as sufficient proof that an* Opsin *can function as a light receptor), we now added data on the sensitivity of the system- see new Fig. 2E-M. The light-dependent effects we see are most sensitive to blue light, which is fully in line with the blue-light sensitivity of medaka* TMT-opsins *and provide no evidence for accessory functions*. *Last, but least, as a matter of principle, we don't think that any scientific publication can provide "*full or definitive answer"s *to scientific questions, but just provide new views and perspectives that help to overall develop our understanding of effects of light on animals further. And in this sense, we think our study provides significant, highly novel and partly unexpected contributions from multiple angles (like the age-dependent effects, the individual behavioral effects and non-additive combined functions, the molecular targets, the photoperiod effects…). This is at present unparalleled and significantly advances our understanding of light effects on physiology and behavior.*

Specific issues

1) The authors do not fully consider potential additional off target effects in their TALENs approach and this may be important.

We sincerely disagree with the judgement of the reviewer as we have been very careful with our controls, which go even beyond what is typical for species that are technically not as advanced as mouse or Drosophila melanogaster. Specifically:
– In contrast to Cas9/Crispr used by most researchers to engineer mutations – and as clearly outlined in the original methods section– all our mutations were generated by TALENs. In comparison to Cas9/Crispr the length of the recognition site that is targeted by TALENs is much longer, because the encoded nucleases attached to the TALE recognition protein are heterodimeric FokI nucleases, which means that two different TALEN pairs have to come together in order to allow for the gene to be cut. The nuclease monomer doesn't function. This already makes "of-target effects" highly unlikely.
– As also already clearly detailed in the original methods section, we outcrossed all generated mutations at least 4 times to the wildtype CAB strain, prior to assaying. In the case of the tmt-opsin1b mutations this had been done already even 13 times (as the strains were already maintained for longer). Again, this is very much up to (and even beyond) common standards to clear up possible back-ground effects for genetic mutations (and there are many well-published manuscripts that do not even come close to these standards, nevertheless it is clear that their results are valuable, e.g. for medaka Nakayama T et al. 2019, doi: 10.1038/s41559-019-0866-6).
– As also clearly mentioned through-out the text we always used direct relatives of the single mutants (i.e. either siblings or cousins) as wt comparisons, the logic being that any still remaining possible off-target effects should occur in the wt siblings/cousins with a similar probability as they would in the animals carrying the actual mutation (unless the off-target mutation was very closely genetically linked- then see arguments further below).
For double mutants we always compared to each of the corresponding wt siblings/cousins. This is why there are two different wt shown in the analyses. As for the analyses of the double mutants

it is statistically extremely unlikely that there would be consistent behavioral/molecular effects within the double-mutant population that could be caused by the random combination of two independent off-target mutations by crossing, which then would have to been maintained without selecting for it. Genetically, this is not plausible and hence we don't consider this as a possibility.
In summary, by using siblings/cousins, we very well controlled for possible off target effects (in addition to outcrossing and TALENs).
– Furthermore, as also clearly indicated in the manuscript, all analyses with the tmt-opsin1b *mutants were performed with mixtures of at least two independently isolated alleles or as transheterozygous. In such a case consistent phenotypes could only occur if twice the same random off-target mutation happened and such mutations would have to be dominant and then cause specific and light-dependent effects. We consider this as not plausible, and at least as much controlled as reverse genetic functional analyses in many other well-accepted studies. This also controls for off-target mutations that would occur in genetically closely linked regions.*
– In case an off-target mutation was genetically closely linked in tmt-opsin2, for which we have only one allele, we also routinely screen for all our TALENs if there was any sequence that somewhat resembled that TALEN target sequences. There was none (and remember that both TALEN pairs would have to bind in a defined distance to allow the nuclease to be functional).
So, all these points provide strong evidence that we were clearly very careful to avoid any misinterpretation due to possible off target effects.

2) The authors fail to use stimuli that could identify the photopigment involved. TMT opsins have characteristic spectral sensitivity profile. However, the authors do not use monochromatic stimuli or a meaningful range of stimulus intensities in their experiments. Thus, the reader is left to question their functional origin. If the experiments were performed with conventional photobiology protocols the conclusions drawn would have a greater certainty. So for example, no evidence is provided to show that short wavelength light is more effective in the assays, which you might expect if TMT itself were responsible.

In its generality the statement is incorrect. In the original version of the manuscript we already compared blue light with a clear lambda$_{max}$ at about 460nm and compared it to white light with similar total photon counts (previous Fig. 2C,D Supp.Fig.2K,L, spectra were provided in Supp.Fig.2B). These experiments showed that the behavioral phenotype of the fish was less strong under the white light than under the blue light.
However, we see the point raised by the reviewer that testing different close-to-monochromatic light stimuli with as similar photon numbers as possible is important to show that the behavioral differences are indeed mediated by tmt-opsin1b. We performed these experiments for the photokinesis test at juvenile stages (20-22dpf). These analyses clearly show that (at these lower intensity) only blue light mediates statistically different responses between mutant and wildtype and are now presented in the new Figure 2E-M and corresponding text.
We would however also like to point out that our functional hypothesis, i.e. that the opsins *present in inter- and motorneuron networks impact on the brain's information processing, could imply that the complete absence of a specific opsin leads to functional adaptation of the network, an aspect that could explain the tendency of the mutant fish to respond stronger to the red light*

stimulus than their wildtype counterparts (albeit this could also be experimental variation as it is not statistically significant).

3) The authors do not establish that TMT is a short wavelength photopigment in any of the assays they describe – rather their data can only suggest they could have a role. This is a common and fundamental problem with a gene ablation approach in isolation. Ablating a TMT opsin does not in itself prove that it is a photopigment.

As outlined above and also referred to in the text, we have previously clearly shown that the TMT-opsins *we analyze here further in functional detail are alone sufficient to render cells that are not light sensitive to light sensitive cells. In the same publication we also showed that blue light had stronger effects than light of longer wavelengths. This goes together with the also cited publication on absorbance spectra by Sakai et al, which showed (very consistently with our cell analysis data) that medaka* TMT-opsins *are most sensitive to blue light. In our functional characterization in the living animal we now show that these* TMT-opsin *members, which convey blue light sensitivity to neuronal cells in tissue culture show functional alterations in response to blue light. We think that this is ample and highly solid evidence for suggesting that* TMT-opsins *sense light.*

4) They present no convincing evidence for the proposed push-pull differential mechanism that they describe.

We are highly puzzled by this comment of the reviewer. No-where in the entire manuscript are we proposing a "push-pull differential mechanism". The term "push-pull mechanisms"is also not such a highly common term in the Opsin literature, that one could simply assume that we imply such a mechanism, without referring to it.
We find it highly questionable to come up with one's own data interpretation and then criticizing the authors for not providing evidence for this interpretation. This might be interesting to e.g. propose for chromatophore cells in the summer flounder, but as a matter of fact is not what we proposed in our manuscript.
We are proposing modulatory functions of these non-visual opsins on behavior and physiology of medaka, which at least in part is mediated by tmt-opsins *expressed outside the eyes. Importantly, from a functional genetics perspective we show that different* opsins *can function genetically complementary and non-complementary, depending on age and assay, which is highly important if we want to further understand how natural light might impact on fish across ages and which* opsins *provide which contribution. The exact mechanism behind the different complementary and non-complementary effects is at present open- and we were very careful to not overspeculate on this. We thus find this criticism unjustified.*

5) Importantly, given the previously published work, what would happen if the authors were to knock down VA opsin, which they themselves have shown to be co-expressed?

This comment of the reviewer appears rather unjustified to us and is also in partial conflict with other concerns he/she raised above. Above he/she commented that in essence the photopigments

we study are not sufficiently characterized concerning their responses to light, despite existing absorption spectra for one (and one direct medaka paralog), proof of sufficiency of the exact chosen opsins in tissue culture cells and their shown stronger response to blue light. For medaka VA-opsin none of this exist, but nevertheless the same reviewer now requests functional experiments from us for VA-opsin? This is scientifically not very plausible.
Furthermore, as mentioned above in our initial argument about the choices, we agree that VA opsins *would certainly also be interesting to functionally investigate, but also from the technical perspective this request is highly problematic.*
The established way to generate knock-downs in zebrafish and medaka are morpholinos, which are injected into the zygote (or two-cell stage). As morpholinos get diluted with each cell devision, it has well been documented that most specific effects are observed within the first three days of development, albeit sometimes effects can still be found at 5dpf. After that this tool gets unreliable (e.g. Bill et al 2009, doi: 10.1089/zeb.2008.0555.) In medaka, at 5dpf animals are not even hatched and this is well before we analyse our phenotypes. Furthermore, there are several other aspects that make the use of knock-down techniques highly problematic, especially for behavioral analyses. One is for instance the incomplete penetrance of most knock-downs (e.g. see Eisen and Smith 2008, doi: 10.1242/dev.001115). Another knock-down tool -RNAi- has been famously shown to cause severe side-effects in fish models, again not advisable to use, especially when it comes to experiments that include behavioral analyses (Oates et al 2000, doi: 10.1006/dbio.2000.9761). Cas9/Crispr can work highly efficiently in the injected generation, but here we would not use this tool, because how should we control for off-target effect without outcrossing? (A critical point the reviewer brought up him/herself above.).
Last, but not least, generating full scale knock-outs, which would first have to be outcrosses and then incrossed to homozygousity is just completely out of time scale for the revision of a manuscript.
Thus in summary, from both the scientific and the technical perspective, this request is not justified.

Other issues

The sentence in the abstract as follows makes no sense:

"We show that these Opsins interact non-additively, impacting the levels of the preprohormone sst1b, as well as the voltage-gated sodium channel subunit scn12aa and - at least in part independently of the eyes and pineal- the amount of larval day-time rest."

Apparently, it made sense to two other reviewers and was cross-verified by an English native speaker with the relevant functional genetic and behavioral background. However, we acknowledge that this is a rather long and complicated sentence and revised it in the new version of the manuscript, along with other parts of the abstract.

Reviewer #3: Fontinha et al conducted an original study where they report the effect of TMT-opsins (1b and 2) on Medaka brain's expression and behaviour. Using KO generated with TALEs, the authors show that these opsins modulate expression level of a somatostatin (sst1.1b) and the voltage gated sodium channel Nav1.9 (scn12aa) and influence daytime rest in Medaka larvae. This is a very ambitious endeavor.

We thank the reviewers for acknowledging the complexity and difficultness of our study.

Although the authors have a point that this topic of the non-visual functions of brain opsins is very interesting and surely not enough investigated, the manuscript overall does not bring a comprehensive view of the function of these 2 opsins on expression in the brain and relevance to behaviour. More information on anatomy, what is know on circuits expressing the opsins and the propeptide as well as the voltage gated sodium channel, is necessary to reach some understanding on the function of the opsins investigated.

We partly agree with the reviewer and addressed this point in our revised version. Partly, because some of the anatomical information requested is already published (for more details see below).

In addition, the manuscript is written in a convoluted manner, lacking both precision and clarity, which should be corrected in the revised version.

It is a bit perplexing to us that one reviewer (reviewer 1) explicitly mentions the well-written style of our manuscript, while this reviewer criticizes it. What we take from it is that the work we present is certainly multidimensional and complex. We now rephrased and re-shuffled several paragraphs in all sections to make the text better understandable.

Major comments

1. Link between expression and function

As the authors found changes of expression of a pro-peptide and a sodium channel (downregulation of sst1.1b and scn12aa), does the corresponding modulation of these two genes explain the effect seen on the daytime rest ? and if yes, how?
In order to reach a model on the function of these opsins in the Medaka larva, the authors should provide more data to tackle the problem:

a- the authors need to identify anatomically the brain regions where sst1.1b, tmt opsin 1b and scn12aa, tmt opsin 2 are expressed

We agree that knowing the brain regions that are positive of the respective markers is an important step to further understand how the light-dependent impact of the opsins might be connected to behavioral alterations. We would, however, like to point out that for tmt-opsin1b *and* tmt-opsin2 *we already provided very detailed anatomical information about the different*

brain nuclei – and their likely connectivity- in our previous publication Fischer R et al, 2013 doi:10.1371/journal.pbio.1001585, e.g. "We detected tmtops2 in the preoptic area (Figure S7E–F) and tmtops1b and 2 close to the third ventricle in the anterior and ventromedial thalamic nucleus …… we identified tmt-opsins in specific interneuron nuclei: We detected tmtops1b in the inner cellular layer of the olfactory bulb (Figure 3A,B) ……. In addition, tmtops2 and 1b are expressed in the dorsal tegmental nucleus of the midbrain (Figures 3E,F and 4A), a structure receiving telencephalic input in fishes [34]. Tmtops1b is also present in multiple hindbrain cell clusters, part of which we identified to belong to the reticular formation (Figure 4C). Next, tmt-opsins also label at least one motorneuron nucleus: tmtops2 and 1b positive cells are present in the nucleus of the facial nerve located in the hindbrain (Figures 3G,H and 4C). This nucleus innervates the vertebrate facial musculature [34].)…." Etc, etc…. All these annotations were already cross-verified with Prof. Mario Wullimann (LMU Munich, Germany).
Upon the request of the reviewer, we now also performed a similar detailed mapping for the expression domains of sst1b. *The new data are included in Supp.Fig.6 and corresponding text.*
Please note that sst1b is NOT sst1.1. (No-where in our entire manuscript have we called this gene sst1.1.*) In medaka this gene runs by three names-* sst1b, sst3 *and* cort. *We now include a comment on this to make the reader better aware of this.*

We also worked hard to obtain a better understanding of the possible role of sodium channel subunit scn12aa. *Both by* in situ *hybridisations* and *antibody staining it appears that Scn12aa is very broadly present throughout the brain (see new Supplementary Figure 9). For data interpretation, please note that* Scn12aa *is the common ortholog to* Scn5a *and* Scn12a *in amniotes (see phylogenetic tree now included as Supp.Fig.8). We now included a discussion about these additional data in the results and discussion.*

b- based on the anatomical evidence and what is known in other species such as lamprey: do sst1.1 + cells receive projections from tmt opsin 1b + cells?

As mentioned above- Please note that sst1b is NOT sst1.1. and it is not completely clear which member of the lamprey sst/cort family members is the direct ortholog to medaka sst1b. Furthermore, while an Encephalopsin/Tmt-opsin ortholog exists in lamprey, to our best knowledge there are unfortunately no connectivity studies (or any studies beyond its existence) published so far. Placental mammals only possess Encephalopsins (which – albeit related- is a distinct vertebrate Opsin subgroup)*- so using data from mouse* Encephalopsin *to speculate about connectivity could be criticized as too far-fetched, since it is not the correct ortholog. Concerning fish- our study is the most advanced on tmt-opsins (and more generally vertebrate non-visual Opsins expressed in the brain) and downstream targets.*
However, thanks to the request of the reviewer we looked in more detail into the literature on lamprey somatostatin+ cells. This made us aware of the highly interesting colocalization of Somatostatin *and GABA in the CSF-contacting neurons of the hypothalamus, as well as the spinal cord of lamprey. Based on scRNA data from zebrafish neurons (Raj, B et al, 2018, doi: 10.1038/nbt.4103), we found that in zebrafish* gad2 *is present in the same cells that express the sst1b ortholog. We cloned medaka* gad2 *and found that it is broadly expressed, but at least clearly co-expressed with* sst1b *in the medaka tectum, in those groups of cells that also get reduced in* tmt-opsin1b-/- *fish. The situation is less clear for* gad2 *and* sst1b *in the other two regulated domains (posterior hypothalamus and hindbrain), where* gad2 *and* sst1b *do not seem to co-*

localize to the same cells. These new data are now presented in Supp.Fig.7. This suggests that at least the sst1b+ *cells are GABA-ergic and hence inhibitory and that part of the regulation of* tmt-opsin1b *might be via modulation of the inhibitory signal. We now include this aspect into the discussion section of the manuscript. We also include the aspect in the discussion that in the mouse visual system* Somatostatin *itself directly reduces excitatory input to downstream interneurons and speculate on a possible circuit in the fish tectum.*
See text in discussion: "Interestingly, in most cases do tmt-opsin1b mutant fish display enhanced behavioral responses. At present we can only speculate about the contribution of the different tmt-opsin1b+ neurons in the response circuitry …. . "

However, we want to point out that we are rather cautious with speculating too far, because at present we can technically not discern which cells are really required for the alteration in behavior.

c- how do KO mutants for sst1.1 and scn12aa behave similarly to tmt opsin 1b and 2 KO?

Given that knock-downs cannot be done for these kind of experiments (for detailed arguments see comment on reviewer 2's request for a VA-opsin knock-down) and knock-outs have to be outcrossed several times and then incrossed- even if everything worked perfectly well (and there were no lab closures due to Corona-measures), this request goes technically far beyond what is common to ask for in a fair peer review process.
As to sst1b- we even tried for two years unsuccessfully to obtain a mutant. We tried 8 different Crispr-guide RNAs, and while we clearly obtained mosaic mutations with pretty high frequency in the injected generation (and even in the F1 outcross), we were unable to maintain even the heterozygous mutants in subsequent generations. It appears as if the mutation is outcompeted by healthy neighboring cells. In short – we nailed it down to something most likely in the non-coding genomic DNA that appears to be required for some sst1b functionally independent aspects of genome stability. But if this genome region is modified, it seems to be sorted out and not efficiently inherited further. Thus, functionally tackling this gene is not as easy as it might appear.

Concerning scn12aa- as we now show in the manuscript- scn12aa- is very broadly expressed (see new Supplementary Figure 9) and also the common ortholog of amniote scn5a and *scn10a (new Supplementary Figure 8). A full-scale knock-out would likely be not very informative, and a conditional knock-out in medaka is even further beyond the timeline and scope of this manuscript.*

2. Behavioural characterization in the mutants:
a- Is there an effect on spontaneous locomotor activity independent of light levels?
When the authors present the avoidance assay at 7-8dpf, they argue that TMT-opsin 1b is expressed in tectum and reticular formation. A change in the activity of the reticular formation should be seen in basic exploration of Medaka larvae. Did the authors check at the same stage than the avoidance assay, that spontaneous locomotor activity was affected in mutant larvae ?

We agree that this is an interesting aspect raised by the reviewer. Our data do not support a change in the spontaneous locomotor activity, independent of changes in light conditions. This

was already hinted at by the data presented in the previous Fig.1G (now Fig.1G), SuppFig. 2C-J (now SuppFig.4B-I), as well as previous SuppFig. 2K,L (now Fig.2A,B), showing that the changes in behavior are age and light dependent.
These data from the previous assays are now fully supported by our new assay with individual wavelength intensities, showing that consistent with tmt-opsin1b functioning as a blue light receptor, *blue light, but not green or red light (at highly similar photon numbers) results in significant behavioral changes. These new data are now presented in Fig.2E-M.*
We would also like to point out that these behavioral data are consistent with the fact that neither in young larvae, nor in juveniles do we find any changes in cortisol levels prior to the assays (see new data in Fig.2P,Q).
What we speculate is likely the case is that while general baseline locomotor levels are not affected, the fish are more "sensitized" in responding to changes (and not steady-state) in stimuli (and this is dependent on ambient light conditions, and stimulus and age dependent). This is consistent with our hypothesis that tmt-opsins1b *and 2 mediate light-dependent difference in information processing by the brain.*

The authors report that at 9-12 dpf the locomotor activity of tmt 11b mutant was reduced while it was higher than siblings in the 20-22 dpf stage. This discrepancies could be explained by clutch to clutch variability more than real effects of the opsins. How many clutches were used to obtain each behavioral results ? These differences make us wonder if the effect of the mutation is light-dependent or just impacts independently of light the basal locomotor activity of the animal.

Concerning the aspect of the clutch to clutch variability: In contrast to zebrafish medakafish batches are much smaller. Depending on age, size, genetic background females typically produce between 5-40 eggs each day. Our females were typically relatively young, thus the average clutch size was 5-8 eggs. Thus, each single assay has larvae that are from at least three different clutches, in most cases even from different parents and a mixture of the two different alleles (as we set-up multiple mating couples). Furthermore, as already presented in the original manuscript, all these experiments were repeated independently. For instance, we would like to point the reviewer to the previous SuppFig.2C-J, (*now SuppFig.4B-I*), *where we clearly show that the early lower behavioral activity of the* tmt-opsin1b *-/- fish inverses more each day. These are completely independent fish from those shown in previous Fig.2A,B* (now Fig.2N,O). *Similarly, we observed the enhanced activity at later stages several times (see also the newly added Figure2E-M with the lower individual light sensitivities). Hence an explanation due to batch effects can be excluded.*
Concerning the light-independent effects- this aspect belongs to the questions raised earlier in the paragraph- thus see answer on the section above.

b- How can the authors explain that the tmt-opsin 1b mutants respond more in the avoidance assay than the control siblings ? are the cells expressing tmt opsin 1b inhibitory ? How does it work ?

This is certainly an interesting question by the reviewer. As a general statement of caution from our side- we do not think that all tmt opsin 1b+ *cells make up a homogenous cell population.*

However, concerning the cells that by their position in the tectum are most likely involved in processing information of the avoidance assay- these are our thoughts: Tmt-opsin1b+ *tectal cells are positive for ChAT (as we published previously- see Fischer R et al 2013). These cells appear to connect possibly monosynaptically (given the close vicinity and branching of ChAT tectal neurons- see Fischer R et al 2013) to neurons marked by* sst1b/gad2*. tmt-opsin1b effects the production of sst1b on transcript level and it has recently been shown that the neuropeptide* SST (SST-14) *in the mouse visual cortical system* (again co-localized with GABA) *suppresses excitatory inputs to PV+-interneurons in V1. In this paradigm,* SST (SST-14) *leads to enhanced visual gain and orientation selectivity (Song Y-H, 2020 doi: 10.1126/sciadv.aaz0517). Furthermore, it is imaginable that* GABA-*release might also be modulated by* tmt-opsin1 *in its downstream circuity. Thus, there are at least two possibilities how loss of* tmt-opsin1b *might result in increased activity in the tectal circuitry and subsequently behavior. We now also included this aspect in the discussion of the manuscript.*

3. Circuit underlying the behavioural effects:

- Nav1.9 has been involved with pain and excitability of cells while sst1.1 has been identified in numerous brain regions including habenula. Did the author check which brain nuclei are expressing sst1.1b and scn12aa at the same larval stage where the behavior experiments were conducted? Could it be that the same cells express sst1.1b and scn12aa and the tmt-opsins ? Could they be receiving inputs from them? We d like to know more about the circuits being involved with the integration of light and the output behavior, here: daytime rest.

First, concerning the correct orthologies- Nav1.9 *is encoded by scn11a* (*hence also called* SCN11a*), this is NOT the same genes as scn12a(a), which we talk about in the manuscript (no-where in the manuscript did we talk about Nav1.9). As the orthology relationships of the* sodium channel subunits *are complex, we now included a molecular phylogeny (see new Supp.Fig.8). This makes it very clear that there is one ancestral fish/amphibian group (called* scn12a in fish*), which can have paralogs (e.g. in zebrafish:* scn12aa *and* scn12 ab*), but not in medaka (i.e. one* scn12aa *gene). This group duplicated in amniotes, with its members confusingly being called* scn10a *and* scn5a. *This is obviously important to keep in mind for functional comparisons between species. However, while Nav1.9/scn11a is not the right comparison, we did investigate for a possible co-expression between* Scn12aa *and sst1b in medaka. We established an antibody staining for* Scn12aa, *which consistent with the expression pattern of its mRNA appears to be broadly (possibly ubiquitously) expressed. Thus, there is clearly co-expression between scn12aa and sst1b, but it will require significant additional further work that goes beyond this already result-wise densely packed and complex manuscript to really trace the exact circuits that govern these behaviors. We, however, included further thoughts and the new details on* Scn12aa *phylogeny, location and sst1b anatomical positions in the results and discussion part of the manuscript.*

- tmt opsin 2 complements the effect of tmt opsin 1b only at the larval but not juvenile stage: how does this work? where are these opsins express in larval and juvenile stages?

We have already documented in our previous publication (Fischer R et al 2013) that the expression patterns of both tmt-opsin1b *and* 2 *do not appear to change visibly between stages immediately after hatching and adults brains. Hence, we do not think that this alteration in the behavior is due to changes in* tmt-opsin *expression, but rather other processes in the brain. As we have shown in the original version of the manuscript (SuppFig.2C-J, now SuppFig.4B-I*), *the switch occurs within the first days after hatching (please note that medaka hatch when they are already fully freely swimming larvae).*
We agree that the point raised by the reviewer is highly interesting and hence provide now more possible explainations for it in the discussion. In brief, based on published data from other vertebrates (including humans) we think that it is more likely that the switch occurs due to ontogenic changes in the transmitter systems. For details see discussion: "We also showed that the response differences present in tmt-opsin1b versus wt fish, as well as the effects of the tmt-opsin1b/tmt-opsin2 double mutants are strongly age-dependent. … ."

Overall we need more precision in the information (anatomical identification of cells expressing, knowledge on the function of the nuclei targeted, ..) to reach a comprehensive view. As it is the study provides only scattered information, which are not helping us understand how brain opsins modulate behaviour.

We think that our revised version now includes substantial amounts of additional data, as well as an improved introduction and discussion that provides significant advance in our understanding of non-visual Opsin function. As probably all significant scientific studies our work, however, does not only provide significant new insights, but also opens up new questions, which offer food for thought and stimulate experimental designs in the future.

Minor comments

- Intro 2nd para - ref 2 to 9 : detail which behaviour and how the opsin contribute with more details and information, this paragraph is key for our understanding and it lacks content in its current form.

We provided more detail in this paragraph, while by the same time trying to keep it focused.

- Intro 5th para ref 19-24: provide more information on the opsin investigated and the actual effect on behavior with more precision again.

We again provided more detail in this paragraph, while by the same time trying to keep it focused.

- In the last paragraph of the introduction, the authors refer to the voltage-gated scn12aa as a neurotransmitter receptor for Nav1.9, which is completely wrong : ?

We think the reviewer refers the sentence: "Our work suggests that these Opsins provide non-redundant environmental light information to the fish, by this modulating a neurohormone and transmitter receptor, as well as behavior." We thank the reviewer for pointing out the mistake in this sentence, as Scn12aa *is of course no transmitter receptor, but the subunit of a voltage-gated sodium channel (as correctly referred to through-out the other sections of the manuscript). We corrected this.*
As mentioned above, please note that scn12aa does not encode Nav1.9*. (see Supplementary Fig. 8*)

- Results: convoluted writing, effects should be clearly explained specifying direction and amplitude in every section.

As mentioned above, it is always a bit problematic to deal with such kind of comments in manuscripts if one reviewer states that the manuscripts is well-written, while another criticises. While revising, we majorly re-wrote large sections of the manuscript and took particular care to improve the explanations in each section (while by the same time trying to balance this with the length of the whole manuscript).
